# Prediction and prognosis of reintubation after surgery for Stanford type A aortic dissection

**DOI:** 10.3389/fcvm.2022.1004005

**Published:** 2022-10-10

**Authors:** Xingxing Yao, Jin Wang, Yang Lu, Xiaofan Huang, Xinling Du, Fuqiang Sun, Yangchao Zhao, Fei Xie, Dashuai Wang, Chao Liu

**Affiliations:** ^1^Department of Cardiovascular Surgery, The First Affiliated Hospital of Zhengzhou University, Zhengzhou, China; ^2^Department of Cardiology, The Sixth People's Hospital of Luohe, Luohe, China; ^3^Department of Cardiology, The First Affiliated Hospital of Zhengzhou University, Zhengzhou, China; ^4^Department of Cardiovascular Surgery, Union Hospital, Tongji Medical College, Huazhong University of Science and Technology, Wuhan, China; ^5^Department of Extracorporeal Life Support Center, Department of Cardiac Surgery, The First Affiliated Hospital of Zhengzhou University, Zhengzhou, China

**Keywords:** aortic dissection, reintubation, risk factors, predictive model, prognosis

## Abstract

**Background:**

Reintubation is a serious adverse respiratory event after Stanford type A aortic dissection surgery (AADS), however, published studies focused on reintubation after AADS are very limited worldwide. The objectives of the current study were to establish an early risk prediction model for reintubation after AADS and to clarify its relationship with short-term and long-term prognosis.

**Methods:**

Patients undergoing AADS between 2016–2019 in a single institution were identified and divided into two groups based on whether reintubation was performed. Independent predictors were identified by univariable and multivariable analysis and a clinical prediction model was then established. Internal validation was performed using bootstrap method with 1,000 replications. The relationship between reintubation and clinical outcomes was determined by univariable and propensity score matching analysis.

**Results:**

Reintubation were performed in 72 of the 492 included patients (14.6%). Three preoperative and one intraoperative predictors for reintubation were identified by multivariable analysis, including older age, smoking history, renal insufficiency and transfusion of intraoperative red blood cells. The model established using the above four predictors showed moderate discrimination (AUC = 0.753, 95% CI, [0.695–0.811]), good calibration (Hosmer-Lemeshow χ^2^ value = 3.282, *P* = 0.915) and clinical utility. Risk stratification was performed and three risk intervals were identified. Reintubation was closely associated with poorer in-hospital outcomes, however, no statistically significant association between reintubation and long-term outcomes has been observed in patients who were discharged successfully after surgery.

**Conclusions:**

The requirement of reintubation after AADS is prevalent, closely related to adverse in-hospital outcomes, but there is no statistically significant association between reintubation and long-term outcomes. Predictors were identified and a risk model predicting reintubation was established, which may have clinical utility in early individualized risk assessment and targeted intervention.

## Introduction

Reintubation is one of the commonly performed surgical procedures for patients with cardiopulmonary insufficiency and consciousness dysfunction after cardiovascular surgery, which is closely related to various adverse outcomes, prolonged hospital stay and increased medical costs (1–8). The reintubation rates reported in previous literature varied considerably due to differences in surgical populations across studies (9–13). Compared with other types of surgery, the incidence of reintubation after Stanford type A aortic dissection surgery (AADS) is relatively higher in the literature, ranging from 7.8 to 20.6% (13–16).

Several studies focused on postoperative reintubation have been conducted and some risk factors have been reported in the literature, such as advanced age and smoking history (4, 11). However, none of those previous studies were designed specifically for patients undergoing AADS or completed in this population. Moreover, no previous studies constructed convenient and practical tools such as nomogram and online risk calculator in this field, which may greatly facilitate the clinical application of prediction model. Our understanding of the risk factors for reintubation after AADS is limited, and there is an urgent need to construct a credible, convenient and practical risk prediction model. In addition, although the relationship between reintubation and in-hospital outcomes have been reported in some previous studies, the relationship between reintubation and long-term prognosis has never been deeply explored or reported so far. It remains unclear whether reintubation after AADS adversely affects the long-term prognosis of patients after discharge.

The objectives of this study were first to identify significant predictors for reintubation in patients undergoing AADS and develop a risk prediction model; and second to deeply explore the relationship between reintubation and in-hospital outcomes and long-term prognosis by univariable and propensity score matching analysis.

## Materials and methods

### Ethical statement

This study was conducted in accordance with the ethical statement of the Declaration of Helsinki, and was approved by The Ethics Committee of Tongji Medical College of Huazhong University of Science and Technology (IORG No. IORG0003571). Written informed consent was waived due to the observational nature of this study.

### Study population

Consecutive adult patients who underwent AADS in a single institution between January-2016 and December-2019 were enrolled. The exclusion criteria of this study were: (1) age < 18 years; (2) time from onset to surgery exceeded 14 days; (3) history of mechanical ventilation within 14 days before surgery; (4) history of organ transplantation, immunosuppression, or immune deficiency; (5) intraoperative or early postoperative death; (6) incomplete medical records.

### Data collection

Clinical data in the hospital were collected through the electronic medical records management system of the hospital. Preoperative factors analyzed in this study included sex, age, body mass index, smoking history, drinking history, hypertension, diabetes mellitus, pulmonary emphysema, chronic bronchitis, cerebrovascular disease, peripheral vascular disease, gastrointestinal tract disease, renal function, atrial fibrillation, general surgical history, cardiac surgery history, New York Heart Association class, pericardial effusion, pulmonary artery hypertension, diameters of the right atrium, right ventricle, left atrium and left ventricle, left ventricular ejection fraction, red blood cell count, hemoglobin, white blood cell count, platelet count, serum creatinine, urea nitrogen, albumin, and globulin. Operative factors included combined surgical types, cardiopulmonary bypass time, aortic cross clamp time, deep hypothermic circulatory arrest, and intraoperative transfusion of red blood cells (RBCs). For clinical factors with multiple measurements, the last measurement result before surgery was used in the analysis.

### Definitions of important variables

Patients' BMI was calculated by dividing their weight in kilograms by their height in meters. Smoking history was defined as previous daily or current smoking. Drinking history was defined as previous alcohol consumption (consumption or >140 g/week >20 g/day) once a week or more over a year or current alcohol consumption. Hypertension was defined based on previous hypertension diagnosis, blood pressure ≥140/90 mmHg, or antihypertensive medication use. Diabetes mellitus was defined based on previous diabetes mellitus diagnosis, diabetic medication use, random glucose ≥11.1 mmol/L, or fasting glucose ≥7.0 mmol/L. Chronic bronchitis was clinically defined by the presence of chronic productive cough or based on imaging findings. Renal insufficiency was defined based on previous diagnosis of renal insufficiency or serum creatinine higher than 110 μmol/L.

### Endpoints and outcome events

In this study, the primary endpoint was postoperative reintubation in patients undergoing AADS. The indications for reintubation included: (1) airway obstruction, progressive aggravation of dyspnea, respiratory failure, weak or stopped spontaneous breathing; (2) malignant arrhythmia, hemodynamic instability, heart failure, cardiogenic shock, cardiac arrest; (3) severe agitation, disturbance of consciousness, loss of consciousness; (4) poor oxygenation, severe respiratory acidosis, refractory hypoxemia; (5) multiple organ dysfunction, requiring treatment with reintubation; and (6) accidental removal of endotracheal intubation while extubation conditions were not reached.

The indications for extubation included: (1) Patients who were fully awake and can follow simple instructions such as opening their eyes, sticking out their tongue, and moving their limbs; (2) stable hemodynamics without low-cardiac output syndrome or myocardial ischaemia, and without significant inotrope support; (3) normothermia; (4) activated coagulation time is normal with no mediastinal bleeding; (5) Muscular strength in accordance with movement of limbs and spontaneous ventilation adequate to maintain arterial oxygen saturation over 95% with 50% FiO2 and end-tidal carbon dioxide below 50 mmHg.

The secondary endpoints in hospital included the length of mechanical ventilation, postoperative pneumonia, tracheostomy, readmission to intensive care unit (ICU), the length of ICU stay, the length of hospital stay, and in-hospital mortality.

The follow-up data of long-term prognosis after discharge were obtained by outpatient surveillance and telephone interviews, included stroke, myocardial infarction, dizziness, limb mobility impairment, all-cause readmission, dissection-related readmission, all-cause death, and dissection-related death.

### Statistical analysis

Statistical analysis was performed using R software (version 4.0.5, www.R-project.org/) and SPSS (IBM SPSS Statistics 26.0, SPSS Inc., Chicago, IL). Two-tailed *P*-value < 0.05 was considered statistically significant.

Categorical variable was expressed as count (percentage). Normally distributed continuous variable was expressed as mean ± standard deviation, otherwise as median (interquartile range). The Q-Q plot was applied to assess whether continuous variable was normally distributed. For univariable analysis, chi-square test or Fisher's exact test was used for categorical variable, Student's *t*-test was used for normally distributed continuous variable, and Mann-Whitney *U*-test was used otherwise. Variables with *P* < 0.1 in the univariable analysis were further analyzed by a forward stepwise multivariable logistic regression procedure to identify significant predictors. The odds ratio (OR) with 95% confidence interval (CI) of each predictor was calculated. A visual nomogram and an online risk calculator based on those predictors and the logistic regression rule were then constructed.

The assessment of the prediction model was performed in the internal population. Internal validation was performed using bootstrap method with 1,000 replications. Calibration was evaluated by both visual inspection of calibration plot and Hosmer-Lemeshow goodness-of-fit test. Discrimination was evaluated using the area under the receiver operating characteristic (ROC) curve (AUC). Clinical utility was evaluated by decision curve analysis.

When analyzing the association between reintubation and other outcomes, univariable and multivariable regression analysis was used to control for confounders. Factors used in the multivariable analysis included sex, age, body mass index, smoking history, hypertension, diabetes mellitus, pulmonary emphysema, chronic bronchitis, cerebrovascular disease, renal function, atrial fibrillation, cardiac surgery history, New York Heart Association class, pericardial effusion, left ventricular ejection fraction, hemoglobin, white blood cell count, platelet count, albumin, cardiopulmonary bypass time, and intraoperative transfusion of RBCs. Survival analysis was performed using Kaplan-Meier curve and log-rank test.

## Results

### Demographic characteristics

After screening, a total of 492 patients met the inclusion criteria and were included in this study. The average age of these patients was 49.6 ± 11.3 years, 75.6% were male. The rate of reintubation after AADS was 14.6% (72/492). The baseline characteristics and operative variables of the included patients are summarized in Supplementary Table 1.

### Development of the risk prediction model

**Figure 1:**
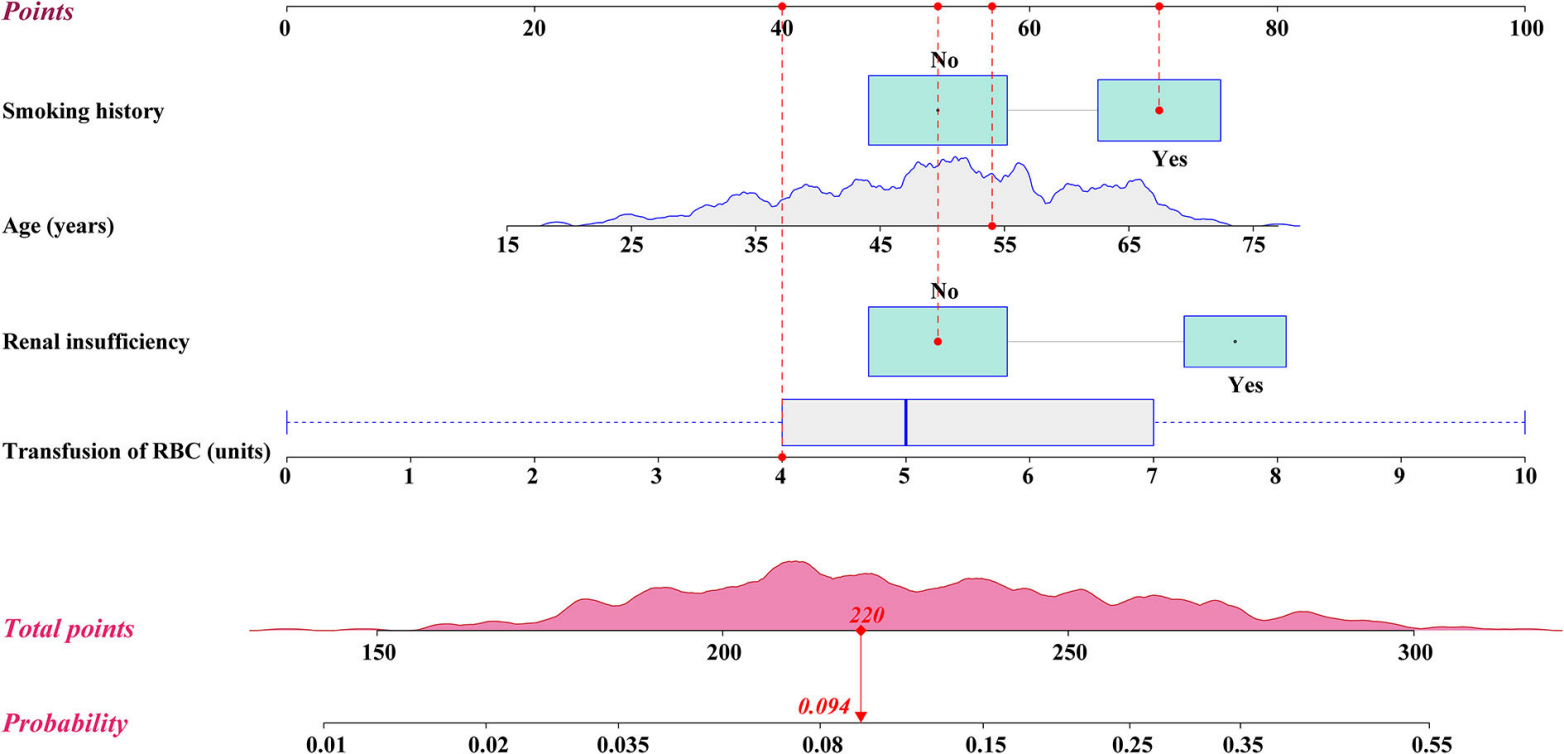
Nomogram for the prediction of postoperative reintubation in patients undergoing AADS. A specific patient was shown to illustrate how to use the nomogram. This was a 54-year-old patient who had smoking history, normal renal function, and was transfused 4 units of RBCs intraoperatively. The individual item point corresponding to each factor is presented at the top, and the total scores were obtained from the sum of the points corresponding to each factor by a red dot. Given values of the 4 predictors, the patient can be intuitively mapped onto the nomogram. It can be clearly seen from the nomogram that the total scores of this patient was 220 points and the corresponding probability of reintubation was 0.094. AADS, Stanford type A acute aortic dissection surgery; RBC, red blood cell.

Univariable analysis was first applied to screen potential predictors for reintubation after AADS and the results are presented in Table 1. Variables with P <0.1 or considered clinically significant were further entered into multivariable logistic regression analysis. Four significant predictors were identified in the final multivariable logistic model, including older age, smoking history, renal insufficiency, and intraoperative transfusion of RBC (Table 2). A visual nomogram based on the logistic rule and the four significant predictors was then established used for the prediction of reintubation after AADS (Figure 1). All the predictors were scaled to 0–100 points based on their regression coefficients, reflecting their relative weight.

**Table 1:** Univariable analysis of possible predictors for reintubation after AADS.

**Characteristic**	**Without reintubation** ***n =* 420 (%)**	**With reintubation** ***n =* 72 (%)**	**χ^2^/Z/t**	***P* value**
Demographics
Male	311 (74)	61 (84.7)	3.798	0.051
Age (years)	49.10 ± 11.39	52.82 ± 10.33	2.594	0.010
Body mass index (kg/m^2^)	25.29 ± 3.65	25.64 ± 3.96	0.743	0.458
Smoking history	174 (41.4)	42 (58.3)	7.132	0.008
Drinking history	148 (35.2)	28 (38.9)	0.357	0.550
Underlying conditions
Hypertension	281 (66.9)	54 (75.0)	1.854	0.173
Diabetes mellitus	18 (4.3)	3 (4.2)	0.002	0.963
Chronic bronchitis	87 (20.7)	19 (26.4)	1.171	0.279
Pulmonary emphysema	19 (4.5)	5 (6.9)	0.776	0.378
Cerebrovascular disease	71 (16.9)	17 (23.6)	1.882	0.170
Peripheral vascular disease	55 (13.1)	12 (16.7)	0.666	0.414
Renal insufficiency	132 (31.4)	41 (56.9)	17.552	<0.001
Gastrointestinal tract disease	35 (8.3)	7 (9.7)	0.152	0.697
Atrial fibrillation	4 (1.0)	0 (0)	0.691	0.406
Cardiac surgery history	28 (6.7)	4 (5.6)	0.125	0.724
General surgery history	83 (19.8)	18 (25.0)	1.034	0.309
New York Heart Association III-IV	35 (8.3)	6 (8.3)	0.000	1.000
Pulmonary artery hypertension	12 (2.9)	2 (2.8)	0.001	0.970
Pericardial effusion	110 (26.2)	23 (31.9)	1.032	0.310
Diameter of the left atrium (cm)	3.5 (3.1, 3.9)	3.7 (3.2, 3.9)	2.110	0.035
Diameter of the left ventricle (cm)	4.8 (4.5, 5.2)	4.8 (4.5, 5.4)	0.558	0.577
Diameter of the right atrium (cm)	3.7 (3.4, 4.0)	3.8 (3.5, 4.0)	0.454	0.650
Diameter of the right ventricle (cm)	3.6 (3.3, 3.9)	3.6 (3.3, 3.9)	0.075	0.940
Left ventricular ejection fraction (%)	62 (60, 65)	62 (60, 65)	0.325	0.745
Laboratory values
White blood cell count (× 10^9^/L)	9.9 (7.5, 12.5)	11.1 (7.9, 13.8)	1.397	0.162
Red blood cell count (× 10^12^/L)	4.2 (3.8, 4.6)	4.2 (3.7, 4.7)	0.098	0.922
Hemoglobin (g/l)	128 (114, 139)	132 (111, 142)	0.675	0.500
Platelet count (× 10^9^/L)	162 (128, 207)	147 (117, 181)	2.344	0.019
Serum creatinine (μmol/L)	79.4 (65.3, 108.2)	90.9 (69.4, 144.9)	2.361	0.009
Serum urea nitrogen (mmol/L)	6.0 (4.9, 7.7)	7.1 (5.7, 9.9)	3.773	<0.001
Serum albumin (g/L)	38.0 (35.0, 41.0)	37.0 (34.0, 40.0)	2.030	0.042
Serum globulin (g/L)	25.6 (23.0, 28.3)	24.7 (21.9, 28.4)	1.063	0.288
Surgical Types			1.272	0.866
Isolated AADS	275 (65.5)	47 (65.2)		
Combined valve surgery	93 (22.1)	17 (23.6)		
Combined coronary artery bypass grafting	22 (5.2)	4 (5.6)		
Combined valve and coronary surgery	23 (5.5)	4 (5.6)		
Combined other types of cardiac surgery	7 (1.7)	0 (0.0)		
Cardiopulmonary bypass time (minutes)	210 (174, 254)	230 (181, 275)	1.934	0.053
Aortic cross clamp time (minutes)	119 (96, 147)	126 (103, 152)	1.477	0.140
Deep hypothermic circulatory arrest	248 (59.0)	42 58.3)	0.013	0.909
Transfusion of red blood cells (units)	4 (4, 7)	7 (5, 9)	5.584	<0.001

AADS, Stanford type A acute aortic dissection surgery.

**Table 2:** Multivariable analysis of significant predictors for reintubation after AADS.

**Characteristic**	**Coefficient**	**OR (95% CI)**	***P* value**
Age (years)	0.030	1.031 (1.006–1.056)	0.015
Smoking history	0.536	1.709 (0.998–2.927)	0.051
Transfusion of red blood cells (units)	0.300	1.350 (1.182–1.542)	<0.001
Renal insufficiency	0.720	2.054 (1.179–3.578)	0.011
Intercept	−5.628	0.004	<0.001

AADS, Stanford type A acute aortic dissection surgery; CI, confidence interval; OR, odds ratio.

The probability of reintubation after AADS can be easily predicted on the nomogram by summing the points of all the predictors. Older patients who have smoking history, renal insufficiency, and more intraoperative transfusion of RBC may obtain higher points and have higher risk of reintubation. A specific case is shown in Figure 1. We also created and provided an online risk calculator to predict the probability of reintubation after AADS to facilitate the clinical application (https://reintubation-prediction.shinyapps.io/dynnomapp/).

### Assessment of the risk prediction model

**Figure 2:**
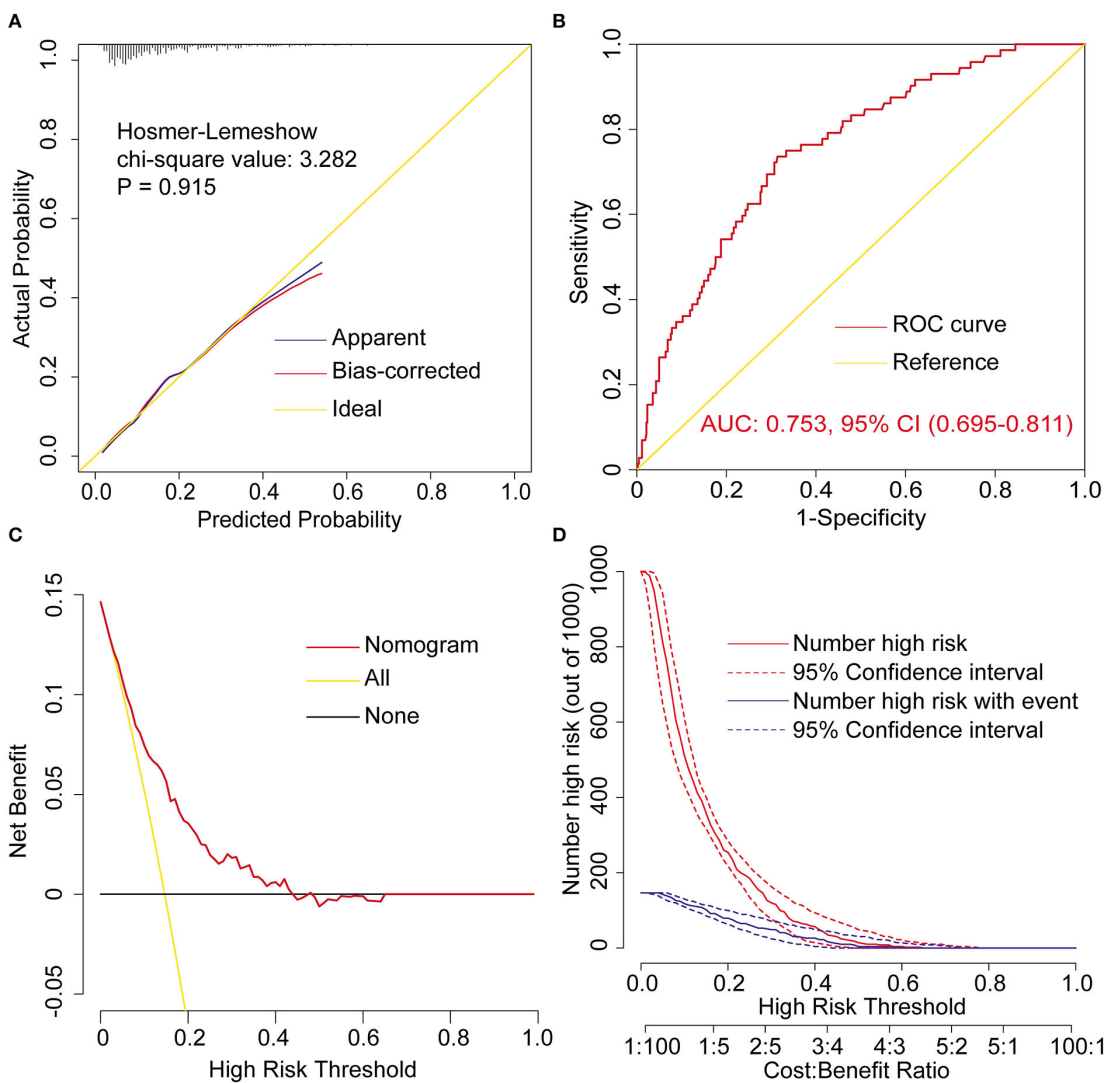
Assessment of the prediction model for postoperative reintubation in patients undergoing AADS. **(A)** Calibration plot and the result of goodness-of -fit test, **(B)** ROC curves and corresponding AUC, **(C)** decision curves, and **(D)** clinical impact curves. AADS, Stanford type A acute aortic dissection surgery; AUC, area under the receiver operating characteristic curve; CI, confidence interval; ROC, receiver operating characteristic curve.

The model was well calibrated by both visual inspection and goodness-of-fit test (Hosmer-Lemeshow χ^2^ value = 3.282, *P* = 0.915, Figure 2A). The model showed moderate discrimination by plotting ROC curve and calculating the AUC (AUC = 0.753, 95% CI, [0.695–0.811], Figure 2B). The model showed remarkable clinical utility by decision curve analysis (Figures 2C,D). The decision and clinical impact curves indicated that compared with treat-none/all strategies, more clinical net benefits could be obtained between the threshold range of 0.05–0.43 when using this model.

### Risk stratification

Based on the nomogram model and clinical practice, a risk stratification procedure was further performed to better facilitate clinical application (Table 3). All the patients were divided into 3 risk groups named low-, medium-, and high-risk group. The cutoff values of the predicted probabilities were respectively 0.1 and 0.3, corresponding to 215 and 251 points on the nomogram. In this study, about forty percent of the patients were divided into low risk group (39.2%), about half of the patients were divided into medium risk group (52.7%), and less than ten percent of the patients were divided into high risk group (8.1%).

**Table 3:** Risk intervals of reintubation based on the nomogram and clinical practice.

**Risk intervals**	**Low risk (<222 points)**	**Medium risk** **(222–267 points)**	**High risk (>267 points)**
Estimated probability (%)	<10	10–30	>30
Estimated probability, % (95% CI)	7.1 (6.8–7.4)	16.9 (16.2–17.5)	36.5 (35.4–37.7)
Observed probability, % (95% CI)	7.3 (3.6–10.9)	17.4 (12.7–22.0)	32.5 (17.3–47.7)
No. of patients (%)	193 (39.2)	259 (52.7)	40 (8.1)

CI, confidence interval.

### In-hospital outcomes

Clinical outcomes in hospital are compared and summarized in Table 4. The overall in-hospital mortality in this study was 9.96% (49/492), with a rate of 4.5% in patients without reintubation vs. 41.7% in those with reintubation (*P* < 0.001). Significantly poorer outcomes with regard to mechanical ventilation, pneumonia, tracheostomy, readmission to ICU, ICU stay and hospital stay were also observed in patients with reintubation in the univariable analysis (Table 4). To deeply reveal the association of reintubation and these outcomes, we further performed multivariable regression analysis. The results showed that after controlling for confounders, there was still a significant association between reintubation and these outcomes.

**Table 4:** In-hospital clinical outcomes in patients with and without reintubation after AADS.

**Variables**	**All patients** ***n =* 492 (%)**	**Without reintubation** ***n =* 420 (%)**	**With reintubation *n =* 72 (%)**	**Unadjusted OR (95% CI)**	***P* value**	*Adjusted OR (95% CI)	*P value
Mechanical ventilation (hours)	63.1 (40.3, 94.9)	59.2 (39.1, 88.2)	105.9 (67.5, 155.7)	1.002 (1.000–1.004)	<0.001	1.001 (1.000–1.003)	0.020
Pneumonia	170 (34.6)	108 (25.7)	62 (86.1)	17.911 (8.869–36.171)	<0.001	11.875 (5.738–24.574)	<0.001
Tracheostomy	55 (11.2)	22 (5.2)	33 (45.8)	15.308 (8.138–28.794)	<0.001	9.984 (5.117–19.480)	<0.001
Readmission to ICU	44 (8.9)	19 (4.5)	25 (34.7)	11.226 (5.752–21.910)	<0.001	7.337 (3.547–15.180)	<0.001
ICU stay (hours)	154.3 (108.1, 254.5)	135.8 (97.9, 201.3)	400.75 (297.0, 571.1)	1.007 (1.005–1.009)	< 0.001	1.006 (1.004–1.008)	<0.001
Hospital stay (days)	21 (17, 27)	21 (17, 26)	29 (19, 39)	1.044 (1.025–1.064)	<0.001	1.025 (1.004–1.045)	0.017
Mortality	49 (10.0)	19 (4.5)	30 (41.7)	15.075 (7.817–29.072)	<0.001	8.999 (4.448–18.206)	<0.001

* Adjusted for sex, age, body mass index, smoking history, hypertension, diabetes mellitus, pulmonary emphysema, chronic bronchitis, cerebrovascular disease, renal function, atrial fibrillation, cardiac surgery history, New York Heart Association class, pericardial effusion, left ventricular ejection fraction, hemoglobin, white blood cell count, platelet count, albumin, cardiopulmonary bypass time, and intraoperative transfusion of RBCs. AADS, Stanford type A acute aortic dissection surgery; CI, confidence interval; ICU, intensive care unit; OR, odds ratio.

### Follow-up outcomes

We performed a long-term follow-up of 443 patients who were successfully discharged from the hospital. The maximum follow-up time was 72 months, the median follow-up time was 40 [31, 51] months, and 32 (7.2%) patients were lost to follow-up. Out-of-hospital information of these patients were obtained through follow-up, including stroke, myocardial infarction, dizziness, limb mobility impairment, all-cause readmission, dissection-related readmission, all-cause death, and dissection-related death, which are summarized in Table 5. In univariable analysis, no significant differences were observed regarding these out-of-hospital events between patients with and without reintubation (Table 5). In multivariable analysis, the association between reintubation and these outcomes remained insignificant after controlling for confounders.

**Table 5:** Long-term follow-up outcomes and comparison in patients with and without reintubation after AADS.

**Variables**	**All patients** ***n =* 443 (%)**	**Without reintubation** ***n =* 401 (%)**	**With reintubation *n =* 42 (%)**	**Unadjusted OR (95% CI)**	***P* value**	*Adjusted **OR (95% CI)**	*P value
Stroke	18 (4.1)	16 (4.0)	2 (4.8)	1.203 (0.267–5.422)	0.810	1.737 (0.289–10.431)	0.556
Myocardial infarction	3 (0.1)	3 (0.7)	0 (0)	-	0.574	-	0.996
Dizziness	34 (7.7)	32 (8.0)	2 (4.8)	0.577 (0.133–2.496)	0.456	0.440 (0.085–2.289)	0.329
Limb mobility impairment	17 (3.8)	15 (3.7)	2 (4.8)	1.287 (0.284–5.830)	0.743	1.527 (0.239–9.754)	0.655
All-cause readmission	116 (26.2)	105 (26.2)	11 (26.2)	1.000 (0.485–2.061)	0.999	0.917 (0.417–2.015)	0.829
Dissection-related readmission	99 (22.3)	88 (21.9)	11 (26.2)	1.262 (0.610–2.612)	0.530	1.218 (0.548–2.711)	0.628
All-cause death	42 (9.4)	37 (9.2)	5 (11.9)	1.329 (0.492–3.589)	0.573	0.788 (0.249–2.496)	0.685
Dissection-related death	35 (7.9)	30 (7.5)	5 (11.9)	1.671 (0.612–4.567)	0.312	1.087 (0.326–3.620)	0.892

* Adjusted for sex, age, body mass index, smoking history, hypertension, diabetes mellitus, pulmonary emphysema, chronic bronchitis, cerebrovascular disease, renal function, atrial fibrillation, cardiac surgery history, New York Heart Association class, pericardial effusion, left ventricular ejection fraction, hemoglobin, white blood cell count, platelet count, albumin, cardiopulmonary bypass time, and intraoperative transfusion of RBCs. AADS, Stanford type A acute aortic dissection surgery.

**Figure 3:**
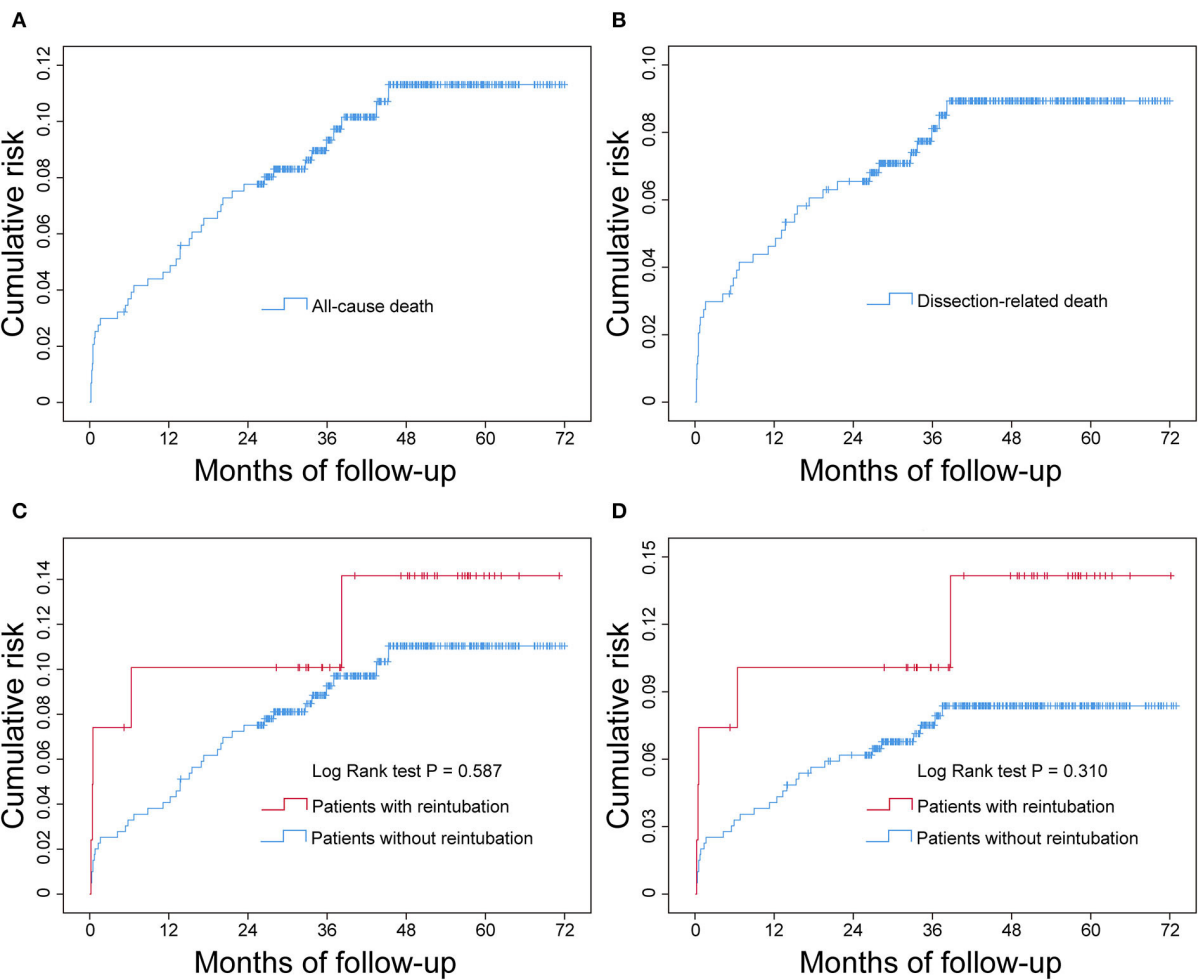
Cumulative all-cause mortality and dissection-related mortality after successful discharge in patients undergoing AADS and the comparison between patients with and without reintubation. The statistical results showed that the difference was not significant between the two groups. **(A)** Cumulative all-cause mortality, **(B)** cumulative dissection-related mortality, **(C)** comparison of cumulative all-cause mortality between patients with and without reintubation, and **(D)** comparison of cumulative dissection-related mortality between patients with and without reintubation. AADS, Stanford type A acute aortic dissection surgery.

To measure the effect of reintubation on the cumulative risk of all-cause death and dissection-related death over time, we plotted Kaplan-Meier curves and performed log-rank test (Figure 3). The results showed that there was a rapid increase in cumulative mortality in the early stage of discharge, followed by a gradual slowdown (Figures 3A,B). Although the cumulative probabilities of both all-cause death and dissection-related death were higher in patients with reintubation by visual inspection of the Kaplan-Meier curves, the differences were not statistically significant between the two groups by log-rank test (Figures 3C,D).

## Discussion

Reintubation is a serious adverse respiratory event after cardiovascular surgery and is closely associated with an increased risk of poor postoperative outcomes (1–8), which was again confirmed by our findings. In this study, the reintubation rate after AADS was 14.6%, and the in-hospital mortality rate was 9.96%, which was similar to the morbidity and mortality reported in the literature. Compared with patients without reintubation, patients who experienced reintubation had more postoperative complications and worse in-hospital outcomes, which further emphasized the need to identify significant predictors and establish a prediction model, so as to achieve the purpose of early prediction, prevention and treatment. However, no statistically significant association between reintubation and long-term outcomes has been observed in patients who were discharged successfully after surgery.

In this study, we identified four significant predictors for postoperative reintubation using clinical data of 492 patients undergoing AADS in a single cardiovascular center, including older age, smoking history, renal insufficiency and the amount of intraoperative transfusion of RBCs. A nomogram and an online risk calculator used to facilitate the prediction of reintubation after AADS was then constructed, which showed moderate discrimination, calibration and clinical utility. To the best of our knowledge, this is the first report that attempts to construct a nomogram and an online risk calculator for reintubation in patients undergoing AADS worldwide, which has certain clinical value and guiding significance.

Predictors for postoperative reintubation varied widely among different literature reports. However, the risk of reintubation has been reported to increase gradually with age in most studies (7, 9, 11, 15), which was consistent with the results of this study. Brovman et al. conducted a biinstitutional study to explore the relationship between early extubation and postoperative reintubation after elective cardiac surgery, finding that older age was an independent risk factor for postoperative reintubation (11). The risk of reintubation increased significantly with age in both coronary artery bypass grafting and aortic valve replacement. Beverly et al. obtained similar results when exploring risk factors for unplanned reintubation after cardiac surgery. In their multivariable analysis, compared to patients under 50 years, the risk of postoperative reintubation increased to 1.41, 2.03 and 3.08 times in patients aged 50–65, 65–80 and over 80 years, respectively (7). Vemuri et al. conducted a large population-based study to explore the effect of patient age on postoperative complication rates following abdominal aortic aneurysm repair in the United States (15). They grouped the population by age and found that the rate of postoperative reintubation increased significantly with increasing age. The rates of postoperative reintubation in patients aged 51–60, 61–70, 71–80, and over 80 years were respectively 3.9, 6.3, 9.3, and 9.5%, and the risk increased to 1.6, 2.3, and 2.7 times, respectively. Therefore, it is necessary to predict the risk of reintubation in advance and take preventive measures to avoid postoperative reintubation in elderly patients.

In the multivariable analysis, smoking history was identified as an independent risk factor for postoperative reintubation, consistent with the results of some previous studies (4, 5, 17). Brovman et al. conducted a retrospective cohort study to determine the frequency, associated risk factors and complications of reintubation in vascular surgery patients, finding that increased age, smoking status and open thoracic and abdominal aorta surgery were independently associated with the increased risk of unplanned reintubation (5). Burton et al. obtained similar results when investigating perioperative risk factors for unplanned reintubation after lung resection (4). In their multivariable regression analysis results, the risk of postoperative unplanned reintubation increased to 1.48 times in patients with smoking history. In addition, previous studies have confirmed that smoking history was closely related to the development of other postoperative respiratory complications, such as hypoxemia and pneumonia, which can significantly increase the risk of postoperative reintubation (18–20). Thus, the relationship between smoking history and the need for postoperative reintubation can be further explained. Recently, Khanna et al. conducted a large-scale study to explore risk factors for pulmonary complications after cardiothoracic surgery, mainly including pneumonia, prolonged mechanical ventilation and reintubation (17). After multivariable regression analysis, they identified 25 independent risk factors, including age, body mass index, smoking, creatinine values, thoracic aortic surgery, and RBC input. Although the results of their analysis focused on the overall pulmonary complications after cardiothoracic surgery, there were some concordances with some of the findings of this study. At the same time, this also confirms and reminds us that smoking is harmful to health, while not smoking or quitting smoking early may have a positive impact on long-term health and the prevention of public respiratory diseases.

Another significant predictor identified by multivariable analysis for reintubation after AADS was renal insufficiency, in agreement with the results of some previous studies (7, 14, 17, 21, 22). In the findings of Beverly et al., chronic kidney disease was another independent risk factor for unplanned reintubation besides age, with a 2.2-fold increased risk compared to patients with normal renal function (7). When studying perioperative risk factors for extubation failure after cardiac surgery, Rady et al. found that blood urea nitrogen levels greater than or equal to 24 mg/dL was an independent risk factor for postoperative reintubation (22). This was similar to the results of Etz et al., who explored the predictors, prevention, and treatment for pulmonary complications after descending thoracic and thoracoabdominal aortic aneurysm repair (14). Rujirojindakul et al. conducted a time-matched, case-control study on anesthetic patients to investigate risk factors for reintubation, finding that creatinine clearance rate was independently associated with postoperative reintubation (21). Compared with patients with creatinine clearance greater than 60%, patients with creatinine clearance of 25–60% and less than 24% had a 2.49-fold and 4.08-fold increased risk of postoperative reintubation, respectively. They believed that this may be related to the fact that renal insufficiency could lead to impaired excretion of anesthetic agents and thus would prolong the duration of the drugs. In addition, renal insufficiency has been reported to be closely related to the occurrence of various postoperative respiratory complications in previous literature reports, which may also be partly responsible for the increased risk of reintubation after AADS (18–20).

The amount of intraoperative transfusion of RBCs was another significant predictor for reintubation after AADS identified by multivariable logistic regression analysis. The risk of postoperative reintubation gradually increased with the increase of RBCs infusion, consistent with previous reports (17, 22). Although blood transfusion is routine and can be life-saving in traditional cardiovascular surgery, increasing evidence indicates that massive blood transfusion is closely related with the occurrence of various postoperative complications and adverse outcomes (23–26). In the findings of Rady et al., transfusion of more than 10 units of blood products was an independent risk factor for perioperative extubation failure in cardiac surgery (22). In the findings of Khanna et al., intraoperative transfusion of RBCs and other blood products were identified as independent risk factors for postoperative pulmonary complications, with the risk increased to 1.81 and 1.52 times, respectively, and the risk increased to 1.05 times with each more 500 ml transfusion of intraoperative blood and fluids (17). Previous studies have demonstrated that massive blood transfusion can cause multiple respiratory complications such as hypoxemia and pneumonia, which is associated with inflammatory response, decreased oxygen-carrying capacity and changes in immune function (27–31). The risk of reintubation may significantly increase with gradually deteriorated cardiopulmonary function and multiple organs of the patients (32). In recent years, increasing evidence has shown that restrictive transfusion strategies are safe and effective, which are also recommended by clinical practice guidelines (33–35).

Several other predictors for postoperative reintubation have also been reported in previous reports but were not identified as predictors in our analysis, including sex, body mass index, chronic lung disease, cardiac surgery history, cardiac function, and cardiopulmonary bypass time (4, 5, 7, 11, 17, 36). This may be due to the differences in the study population and the type of surgery. The large differences in predictors for reintubation after AADS and other surgical types further revealed the specificity of AADS. Therefore, it may not be appropriate to use existing risk prediction models developed for other types of surgery to predict the risk of reintubation after AADS, which also highlighted the necessity of this study.

Some postoperative variables such as the duration of mechanical ventilation and pneumonia were identified as independent risk factors for postoperative reintubation and included in the final model in some studies (11), but we only included preoperative and intraoperative variables for analysis and for the construction of the risk prediction model. Endotracheal intubation operation can damage the defense mechanism of respiratory system, and the risk of various postoperative respiratory complications may increase significantly with the prolongation of mechanical ventilation time (37, 38). Undoubtedly, endotracheal tube should be removed and the spontaneous breathing should be resumed when conditions permit (39, 40). However, we did not include the duration of mechanical ventilation as a predictor in our multivariable analysis because this was a postoperative variable and cannot be obtained at an early stage in some patients. The purpose of early prediction cannot be achieved if these postoperative variables were included in the model.

Extubation at an optimal time after surgery may significantly improve the prognosis of patients undergoing high-risk operations such as cardiovascular surgery, where a lot of exploration and research has been conducted in this field (41, 42). Balancing the relationship between prolonged mechanical ventilation and the risk of reintubation may have a profound impact on patient outcomes. The analysis results of this study showed that after controlling for confounders by multivariate analysis, the risk of multiple in-hospital adverse outcome events remained significantly higher in the reintubation group. Therefore, a more stringent reintubation strategy should be implemented to reduce unnecessary reintubation for low-risk patients. For high-risk patients, it may be effective to reduce postoperative cardiopulmonary complications and thus reduce the risk of reintubation by strengthening preoperative expectorant, oxygen therapy, respiratory function exercise, nutrition and cardiac function, shortening intraoperative operation time, reducing unnecessary blood transfusion, implementing more reasonable fluid and drug strategies, and paying more attention to their vital signs and taking timely treatment measures (43–48). The model established in this study may have an important role in personalized risk assessment and early prevention. Implementing appropriate preventive measures for high-risk patients identified by the risk prediction model may obtain more clinical net benefits.

For patients who were successfully discharged after surgery, reintubation showed no significant association with postoperative all-cause and dissection-related deaths in the whole population analysis. The difference in the outcomes between the two groups remained statistically insignificant after controlling for confounders by multivariate analysis. This finding may also have certain guiding value for doctor-patient communication and clinical practice.

Several limitations existed in this study. First, this was a single-center small-sized exploratory study, which may limit the generalizability of the findings. However, the conclusions may be more reliable due to the fact that we adopted the same strict and identical indications for reintubation in our center, which may significantly reduce heterogeneity. Second, some potential predictors that may associate with postoperative reintubation were not included in our analysis, such as the use of some blood indicators and drugs (10). Nevertheless, the model established using the four predictors indicated reasonable discrimination, calibration and clinical usefulness. Third, the primary endpoint of this study was reintubation, but the total number of reintubation events was small, especially after propensity score matching, which may have some impact on the findings. Multicenter, large sample size studies and longer follow-up are needed to deeply explore the relationship between reintubation and clinical outcomes in future work.

## Conclusions

The requirement of reintubation after AADS is prevalent, closely related to adverse in-hospital outcomes, but there is no statistically significant association between reintubation and long-term outcomes. To our knowledge, this study represents the first attempt to construct a nomogram and an online risk calculator for reintubation in patients undergoing AADS worldwide, and the first report involving the relationship between reintubation and associated in-hospital and long-term outcomes, which may have certain clinical value and guiding significance. In this study, four significant predictors for reintubation after AADS were identified, including older age, smoking history, renal insufficiency and intraoperative transfusion of RBCs. The model established using the four predictors showed moderate discrimination, good calibration and clinical utility. Three risk groups were identified as low-, medium- and high-risk groups on the basis of the model and clinical practice. These findings may have clinical utility in early individualized risk assessment, informed decision-making and targeted interventions.

## Data availability statement

The raw data supporting the conclusions of this article will be made available by the authors, without undue reservation.

## Ethics statement

The studies involving human participants were reviewed and approved by the Ethics Committee of Tongji Medical College of Huazhong University of Science and Technology. Written informed consent for participation was not required for this study in accordance with the national legislation and the institutional requirements.

## Author contributions

CL, FX, DW, and YZ: conception and design. XH and XD: administrative support. XY, DW, JW, and FS: provision of study materials or patients. XY, DW, YL, and XH: collection and assembly of data. XY, DW, and YL: data analysis and interpretation. All authors contributed to manuscript writing and final approval of manuscript.

## Funding

This work was supported by the National Natural Science Foundation of China (Grant No. 81800413), the Key Scientific Research Projects of Henan Higher Education Institutions (Grant No. 20A320036), and Key R&D and Promotion Projects in Henan Province (Grant No. 202102310123).

## Conflict of interest

The authors declare that the research was conducted in the absence of any commercial or financial relationships that could be construed as a potential conflict of interest.

## Publisher's note

All claims expressed in this article are solely those of the authors and do not necessarily represent those of their affiliated organizations, or those of the publisher, the editors and the reviewers. Any product that may be evaluated in this article, or claim that may be made by its manufacturer, is not guaranteed or endorsed by the publisher.

## Abbreviations

AADS: Stanford type A acute aortic dissection surgery
AUC: area under the receiver operating characteristic curve
CI: confidence interval
ICU: intensive care unit
OR: odds ratio
RBC: red blood cell
ROC: receiver operating characteristic curve.
