# Supplementary material for: Prediction and prognosis of reintubation after surgery for Stanford type A aortic dissection
Source: Front Cardiovasc Med. 2022 Oct 10;9:1004005. doi: 10.3389/fcvm.2022.1004005 (PMC9592067; doi:10.3389/fcvm.2022.1004005)
Supplement: Supplementary file 1 [file Table_1.docx]

Supplementary table 1. The baseline characteristics and operative variables of the included patients

| Characteristic | All patients  n = 492 (%) |
| --- | --- |
| Demographics | |
| Male | 372 (75.6) |
| Age (years) | 49.64 ± 11.31 |
| Body mass index (kg/m2) | 25.34 ± 3.70 |
| Smoking history | 216 (43.9) |
| Drinking history | 176 (35.7) |
| Underlying conditions | |
| Hypertension | 335 (68.1) |
| Diabetes mellitus | 21 (4.3) |
| Chronic bronchitis | 106 (21.5) |
| Pulmonary emphysema | 24 (4.9) |
| Cerebrovascular disease | 88 (17.9) |
| Peripheral vascular disease | 67 (13.6) |
| Renal insufficiency | 173 (35.2) |
| Gastrointestinal tract disease | 42 (8.5) |
| Atrial fibrillation | 4 (0.8) |
| Cardiac surgery history | 32 (6.5) |
| General surgery history | 101 (20.5) |
| New York Heart Association III-IV | 41 (8.3) |
| Pulmonary artery hypertension | 14 (2.8) |
| Pericardial effusion | 133 (27.0) |
| Diameter of the left atrium (cm) | 3.5 (3.1, 3.9) |
| Diameter of the left ventricle (cm) | 4.8 (4.5, 5.2) |
| Diameter of the right atrium (cm) | 3.7 (3.5, 4.0) |
| Diameter of the right ventricle (cm) | 3.6 (3.3, 3.9) |
| Left ventricular ejection fraction (%) | 62 (60, 65) |
| Laboratory values | |
| White blood cell count (× 109/L) | 10.0 (7.5, 12.7) |
| Red blood cell count (× 1012/L) | 4.2 (3.8, 4.6) |
| Hemoglobin (g/l) | 128 (114, 139) |
| Platelet count (× 109/L) | 159 (126, 204) |
| Serum creatinine (μmol/L) | 80.9 (65.8, 112.0) |
| Serum urea nitrogen (mmol/L) | 6.2 (5.0, 7.9) |
| Serum albumin (g/L) | 37.8 (34.9, 40.9) |
| Serum globulin (g/L) | 25.6 (22.6, 28.3) |
| Surgical Types |  |
| Isolated AADS | 322 (65.4) |
| Combined valve surgery | 110 (22.4) |
| Combined coronary artery bypass grafting | 26 (5.3) |
| Combined valve and coronary surgery | 27 (5.5) |
| Combined other types of cardiac surgery | 7 (1.4) |
| Cardiopulmonary bypass time (minutes) | 211 (175, 257) |
| Aortic cross clamp time (minutes) | 120 (96, 147) |
| Deep hypothermic circulatory arrest | 290 (58.9) |
| Transfusion of red blood cells (units) | 5 (4, 7) |

AADS, Stanford type A acute aortic dissection surgery.
